# Detecting transcription of ribosomal protein pseudogenes in diverse human tissues from RNA-seq data

**DOI:** 10.1186/1471-2164-13-412

**Published:** 2012-08-21

**Authors:** Peter Tonner, Vinodh Srinivasasainagendra, Shaojie Zhang, Degui Zhi

**Affiliations:** 1 Department of Electrical Engineering and Computer Science, University of Central Florida, Orlando, FL, 32816, USA; 2 Department of Biostatistics, Section on Statistical Genetics, University of Alabama at Birmingham, Birmingham, AL, 35294, USA

**Keywords:** Ribosomal protein, Pseudogene, Transcription, RNA-seq data

## Abstract

**Background:**

Ribosomal proteins (RPs) have about 2000 pseudogenes in the human genome. While anecdotal reports for RP pseudogene transcription exists, it is unclear to what extent these pseudogenes are transcribed. The RP pseudogene transcription is difficult to identify in microarrays due to potential cross-hybridization between transcripts from the parent genes and pseudogenes. Recently, transcriptome sequencing (RNA-seq) provides an opportunity to ascertain the transcription of pseudogenes. A challenge for pseudogene expression discovery in RNA-seq data lies in the difficulty to uniquely identify reads mapped to pseudogene regions, which are typically also similar to the parent genes.

**Results:**

Here we developed a specialized pipeline for pseudogene transcription discovery. We first construct a “composite genome” that includes the entire human genome sequence as well as mRNA sequences of real ribosomal protein genes. We then map all sequence reads to the composite genome, and only exact matches were retained. Moreover, we restrict our analysis to strictly defined mappable regions and calculate the RPKM values as measurement of pseudogene transcription levels. We report evidences for the transcription of RP pseudogenes in 16 human tissues. By analyzing the Human Body Map 2.0 study RNA-sequencing data using our pipeline, we identified that one ribosomal protein (RP) pseudogene (PGOHUM-249508) is transcribed with RPKM 170 in thyroid. Moreover, three other RP pseudogenes are transcribed with RPKM > 10, a level similar to that of the normal RP genes, in white blood cell, kidney, and testes, respectively. Furthermore, an additional thirteen RP pseudogenes are of RPKM > 5, corresponding to the 20–30 percentile among all genes. Unlike ribosomal protein genes that are constitutively expressed in almost all tissues, RP pseudogenes are differentially expressed, suggesting that they may contribute to tissue-specific biological processes.

**Conclusions:**

Using a specialized bioinformatics method, we identified the transcription of ribosomal protein pseudogenes in human tissues using RNA-seq data.

## Background

Pseudogenes are “fossil” copies of functional genes that have lost their potential as DNA templates for functional products [1-6]. While the definition of pseudogenes is still somewhat fuzzy, most of them are defined operationally by bioinformatics criteria, e.g., genomic scans of signatures of homology to known genes. Ribosomal protein (RP) pseudogenes represent the largest class of pseudogenes found in the human genome: over 2000 ribosomal protein pseudogenes are identified by bioinformatics scan of genomic sequence [5].

These pseudogenes are commonly thought to be non-functional due to the lack of promoters and/or the presence of loss of function mutations. Indeed, the vast majority of these pseudogenes either carry dysfunctional mutations such as in-frame stop codons, or lack of proper regulatory sequences, such as promoters, mTOP signals, and first introns [7]. Interestingly, three RP pseudogenes, with 89%-95% sequence identity to their parent (progenitor) RP genes, were found to be transcribed and seem to be functional, by a bioinformatics scan of cDNA and expression sequence tag (EST) databases and confirmation by PCR and Northern blot [8]. A genome-wide bioinformatics scan identified over 2000 potential pseudogenes [5]. Moreover, it was found [9] that the six RP pseudogenes shared at syntenic loci between the human and the mouse genomes are more conserved than other RP pseudogenes.

However, data were lacking to experimentally validate pseudogene expression. It is unclear from the literature whether the reported cases are merely anecdotal or that pseudogenes do play some cellular roles. This is largely hindered by the lack of methods for the identification of pseudogenes transcription. The traditional method of transcriptome profiling, gene expression microarray, is not sensitive in distinguishing transcripts among very similar gene sequences.

Recent advancements of next-generation sequencing allow for direct massive transcriptome sequencing (RNA-seq), and thus providing unprecedented insights into all transcribed sequences. For example, RNA-seq has been applied to detect complex transcriptional activities such as alternative splicing [10,11] and allelic-specific expression [12]. Recently, RNA-seq has been applied to reveal RNA editing events [13]. However, to the best of our knowledge, there were yet no attempts to detect the transcription of pseudogenes in RNA-seq data. The main challenge for pseudogene identification in RNA-seq data is the difficulty of high fidelity read mapping. Because sequences of pseudogenes are highly similar to the sequences of the mRNAs of the parent genes, specialized read mapping methods are required to detect reads unambiguously generated from pseudogenes.

In this study, we conduct a bioinformatics analysis of pseudogene expression using RNA-sequencing data of 16 human tissues of the Illumina Human Body Map 2.0 project. We first describe our new computational pipeline for detecting pseudogene expression that disentangles sequencing reads of pseudogenes from those of the parent genes, with consideration of possible mismatches due to SNPs and RNA-editing. This is followed by a description of our findings and a discussion of their implications.

## Results

### Illumina Human Body Map 2.0 RNA-seq data

The Human Body Map 2.0 Project by Illumina generated RNA-seq data for 16 different human tissues (adipose, adrenal, brain, breast, colon, heart, kidney, liver, lung, lymph node, ovary, prostate, skeletal muscle, testes, thyroid, and white blood cells). In our analysis we used the 75 bps single read data, with one lane of HiSeq 2000 data per tissue. Standard mRNA-seq library preps were used to extract poly-A selected mRNAs.

### Discovery of pseudogene transcription in RNA-seq data

Our primary goal is to detect transcriptional activities of any of the 1709 processed RP pseudogenes. In addition, we also want to provide a preliminary quantification of their level of transcription.

We developed a novel bioinformatics approach for detecting the subtle signals of pseudogene expression. Briefly, we first compiled a “composite genome” consisting of known RP gene spliced mRNA sequences and the human genome (hg18) [14]. We then mapped RNA-seq reads to the composite genome using Bowtie [15], allowing no mismatches and discarding reads mapped to more than one locus. Thus we ensured that the reads mapped to RP pseudogenes are neither from repetitive regions nor from normal RP genes. On average 89% of the reads aligning to RP pseudogenes can also be mapped to real RP spliced mRNA sequences and are removed when mapped to the composite genome (see Table 1). Furthermore, to remove mapped reads that may be caused by SNPs and RNA-editing, we extended the concept of the mappability (the mappable regions of human genome is called the uniqueome) [16] and masked regions in RP pseudogenes that are duplicated in the composite genome within 4 mismatches over the 75 bps read length. The number of reads we removed from non-unique locations in both the composite genome and hg18 genome can be seen in Table 2. The mappability regions only correspond to the unaltered human genome locations, so all reads mapped to RP gene mRNA sequences in the composite genome are removed during this step. Additionally, the composite genome alignment lacks the reads that mapped both to the unaltered human genome locations and spliced RP gene mRNA sequences as we only retained reads aligning to a single location. With both of these groups of reads removed, the number of reads mapped uniquely in the composite genome is always less than that in the unaltered human genome. Finally, we calculated the transcription levels, as measured by the Reads Per Kilobase per Million reads (RPKM) [11] of all pseudogenes according to the mapped reads in their mappable regions. As a benchmark for normal expression levels, we also aligned reads to an unaltered genome using TopHat and measured FPKM of all RefSeq genes using Cufflinks [17]. The alignment information of reads to the composite genome, and to the unaltered genome (hg18), can be seen in Table 2. Please see Methods for details.

**Table 1:** The number of reads mapped to RefSeq sequences and RP pseudogenes for both the composite genome and the unaltered human genome (hg18) for each tissue

**Tissue**	**RP Pseudogenes**			**RefSeq Sequences**		
	**Composite genome**	**hg18**	**Ratio**	**Composite genome**	**hg18**	**Ratio**
Adipose	708	22613	0.03	22018081	23753007	0.93
Adrenal	2439	22280	0.11	19436010	21074345	0.92
Brain	712	3853	0.18	11585759	12357788	0.94
Breast	1603	15951	0.10	22962845	23060856	1.00
Colon	1066	22948	0.05	21458813	21618780	0.99
Heart	562	2374	0.24	12622175	13502482	0.93
Kidney	1341	5928	0.23	20630500	22278600	0.93
Liver	328	2359	0.14	16689110	17958332	0.93
Lung	1128	11918	0.09	28648503	31274037	0.92
Lymph	1456	14010	0.10	22126747	23998836	0.92
Muscle	459	8705	0.05	25836676	27662752	0.93
Ovary	1501	34608	0.04	21842763	23663484	0.92
Prostate	1280	14976	0.09	30329651	32822472	0.92
Testes	1885	19417	0.10	23356973	25123824	0.93
Thyroid	4064	22250	0.18	24800037	26627946	0.93
White Blood Cells	1095	14256	0.08	25487259	27812790	0.92
**Average**	1351	14902	**0.11**	21864493	23411895	**0.94**

The ratio is calculated by the number of mapped reads from the composite genome over the number of mapped reads from the unaltered human genome (hg18).

**Table 2:** Statistics for each tissue sample

**Tissue**	**Number of reads in the sample**	**Number of reads mapped to the composite genome**	**Number of reads mapped to hg18**	**Number of reads mapped uniquely to the composite genome**	**Number of reads mapped uniquely to the hg18**
Adipose	76,269,225	39,499,413	39,759,404	27,507,408	30,635,265
Adrenal	76,171,569	39,330,423	39,681,721	29,670,249	32,603,673
Brain	64,313,204	21,022,913	21,073,103	15,785,362	17,187,772
Breast	77,195,260	39,355,808	39,568,435	30,955,674	31,072,723
Colon	80,257,757	39,406,195	39,735,900	28,393,105	28,582,515
Heart	76,766,862	29,030,896	29,065,063	17,099,881	19,366,541
Kidney	79,772,393	41,368,095	41,488,175	27,316,562	30,570,744
Liver	77,453,877	26,692,219	26,741,073	18,897,673	20,715,729
Lung	81,255,438	45,996,752	46,211,355	34,401,958	37,862,617
Lymph	81,916,460	41,826,888	42,072,585	30,451,248	33,277,288
Muscle	82,864,636	46,580,440	46,725,392	30,704,302	34,165,828
Ovary	81,003,052	36,922,138	37,385,453	28,184,877	30,861,571
Prostate	83,319,902	47,601,661	47,965,443	36,138,822	39,533,973
Testes	82,044,319	38,852,709	39,069,136	29,115,004	31,927,737
Thyroid	80,246,657	40,546,781	40,785,090	31,137,501	33,939,339
White Blood Cells	82,785,673	38,860,771	39,098,752	28,796,204	31,784,122

The number of reads mapped to the composite genome (which includes spliced ribosomal protein gene sequences) and to the unaltered human genome (hg18), and the number of reads overlapped with uniqueome (“mapped uniquely”) for both are shown. For the composite genome, the number of reads aligning to the entire composite genome and the unaltered hg18 human genome are shown.

### Prevalent transcription of RP pseudogenes

**Figure 1:**
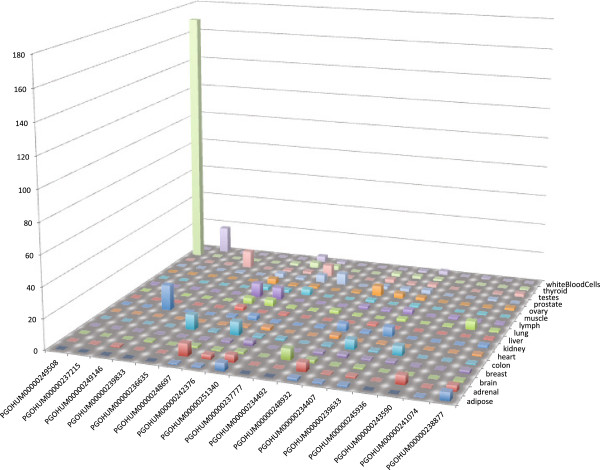
**RPKM of RP pseudogenes.** See Table S1 in Additional file 1 for the complete list.

The expression levels of the top seventeen highly expressed ribosomal protein pseudogenes in 16 human tissues are shown in Figure 1 and Table 3 (See Table S1 in Additional file 1 for complete list for all RP pseudogenes). As expected the majority of pseudogenes have no reads aligning to their sequence. Interestingly, there were some pseudogenes with high expression levels. One RP pseudogene (PGOHUM-249508) is transcribed with RPKM 170 in thyroid. Moreover, three additional RP pseudogenes are transcribed with RPKM > 10. Furthermore, thirteen more RP pseudogenes are of RPKM > 5. We describe pseudogenes with an RPKM > 10 as “highly expressed”, with the understanding that they may be only representing the top 10–15 percentile of all 37,681 RefSeq genes in the Human Body Map 2.0 data set, while RPKM > 5 represents the top 20–30 percentile (see Table 4). Below we discuss these cases in detail.

**Table 3:** RP pseudogenes expression identified in Human Body Map 2.0 RNA-seq data

**pg-id**	**Parent gene**	**Location**	**Tissue with Max RPKM**	**Max RPKM**	**Total RPKM**	**Tissue specificity**	**Reads coverage**
249508	RPL21	chr8:134084035-134084502	Thyroid	170.3	170.6	0.977	0.976
237215	RPL7A	chr17:6984988-6985635	White Blood Cells	17.3	18.0	0.881	0.913
249146	RPS24	chr16:55497947-55498335	Kidney	16.5	17.6	0.855	0.693
239833	RPS11	chr12:63076580-63077044	Testes	11.3	12.5	0.813	0.970
236635	RPL24	chr9:72021934-72022269	Colon	9.7	9.7	1.000	0.358
248697	RPL26	chr16:1953719-1954097	Lymph	9.0	37.9	0.376	0.854
242376	RPS20	chr11:77202016-77202370	Colon	8.6	41.6	0.355	1.00
251340	RPLP1	chr5:151125656-151125993	Testes	7.4	40.1	0.339	0.567
237777	RPL7	chr3:133445060-133445787	Prostate	7.3	8.0	0.819	0.371
234492	RPL10	chr19:9791817-9792131	Brain	7.2	10.8	0.635	0.121
248932	RPL21	chr16:72904438-72904782	Ovary	6.5	34.3	0.343	1.00
234407	RPL39	chr19:58143205-58143330	Colon	6.3	21.3	0.414	1.00
239633	RPL13	chr12:6863424-6864038	Kidney	6.0	27.0	0.365	0.923
245936	RPL6	chr4:66121772-66122638	Colon	5.9	7.2	0.746	0.109
243590	RPL32	chr6:33155206-33155612	Adrenal	5.8	9.2	0.617	1.00
241074	RPL13A	chr2:203093361-203093957	Lung	5.4	9.7	0.571	0.995
238877	RPL11	chr10:89695235-89695766	Adipose	5.2	20.4	0.387	0.847

Expression levels of pseudogenes with their pseudogene ID (pg-id, prefix ‘PGOHUM00000’ omitted) are measured in terms of RPKM. Only pseudogenes with maximum RPKM > 5 are shown. Tissue specificities are measured by the JS divergence [20]. Read coverage is the ratio of pseudogene exon length covered by uniquely mapped reads to the total pseudogene exon length.

**Table 4:** Table of FPKM expression values of RefSeq genes in 16 human tissues

**Tissue**	**FPKM**				**Percentile**				
	**Mean**	**Max**	**Min**	**std dev**	**% > 1**	**% > 2**	**% > 5**	**% > 10**	**% > 15**
Adipose	10.03	17437.80	0	143.53	41.07	32.42	20.62	12.64	8.96
Colon	10.42	17376.90	0	167.07	40.80	31.61	19.67	11.93	8.37
Heart	7.55	17376.90	0	122.87	36.61	27.71	16.09	9.41	6.43
Kidney	9.97	16400.20	0	156.94	45.28	35.93	22.62	13.32	9.23
Liver	14.91	38505.10	0	360.25	33.57	25.10	14.90	8.93	6.48
Lung	16.29	57096.60	0	456.70	44.59	35.08	22.05	13.61	9.72
Lymph	13.41	40919.80	0	337.50	45.77	36.77	23.25	13.70	9.22
Muscle	8.80	16317.70	0	123.85	33.27	25.97	16.38	10.39	7.55
Ovary	13.62	61099.30	0	359.00	46.27	37.60	24.59	15.05	10.37
Prostate	13.72	39039.90	0	300.91	47.82	38.83	25.36	15.68	10.79
Testes	12.76	57096.60	0	325.41	53.34	43.44	28.69	17.85	12.46
Thyroid	12.06	29030.10	0	234.59	46.04	37.25	24.24	15.12	10.74
White Blood Cells	15.42	40919.80	0	366.35	39.25	32.56	22.65	14.87	10.83
All Tissues	12.23	61099.30	0	286.98	42.59	33.87	21.62	13.27	9.32

Percentile columns represent the percentage of RefSeq genes in all tissues with FPKM above a given value.

**Figure 2:**
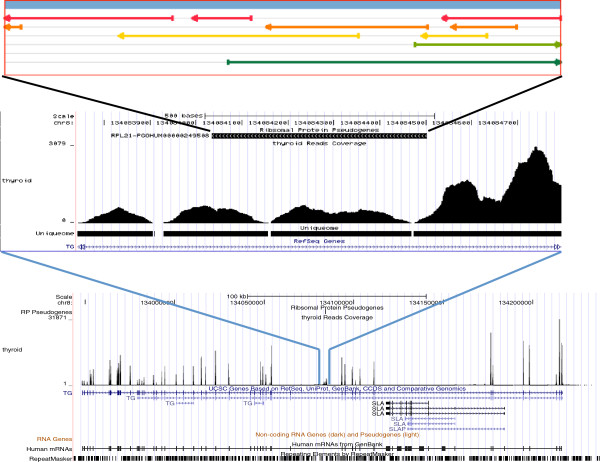
**UCSC browser view of RNA-seq expression of pseudogene PGOHUM-249508 in Thyroid.** RPKM = 170, Tissue Specificity = 0.977. Open reading frames (ORFs) in +1, +2, +3, -1, -2, and −3 are annotated.

**PGOHUM-249508**, an RPL21 pseudogene, is expressed with RPKM = 170 in thyroid (Figure 2). This highest expressed RP pseudogene is located in an intron of the Thyroglobulin (TG) gene. The TG gene is highly and specifically expressed in the same tissue, thyroid, and the gene encodes a glycoprotein that acts as a substrate for the synthesis of thyroxine and triiodothyronine as well as the storage of the inactive forms of thyroid hormone and iodine [18]. The transcription of this pseudogene goes beyond the annotated pseudogene region, but is less than the entire intron region. Although the pseudogene is specifically expressed in the same tissue as TG, the RP coding frame is on the reverse strand of the TG gene. Therefore, it is possible that this pseudogene is on a distinct transcript other than the TG gene. Moreover, according to UCSC genome browser [19], this pseudogene region is only conserved within the primates (between human and the Rhesus monkey), but not in other mammalian and vertebrate lineages. As a side note, the genome browser shows a peculiar conservation pattern between human and the stickleback fish, but it is likely to be an artifact of matching human genomic sequence with the RPL21 gene of stickleback fish.

**Figure 3:**
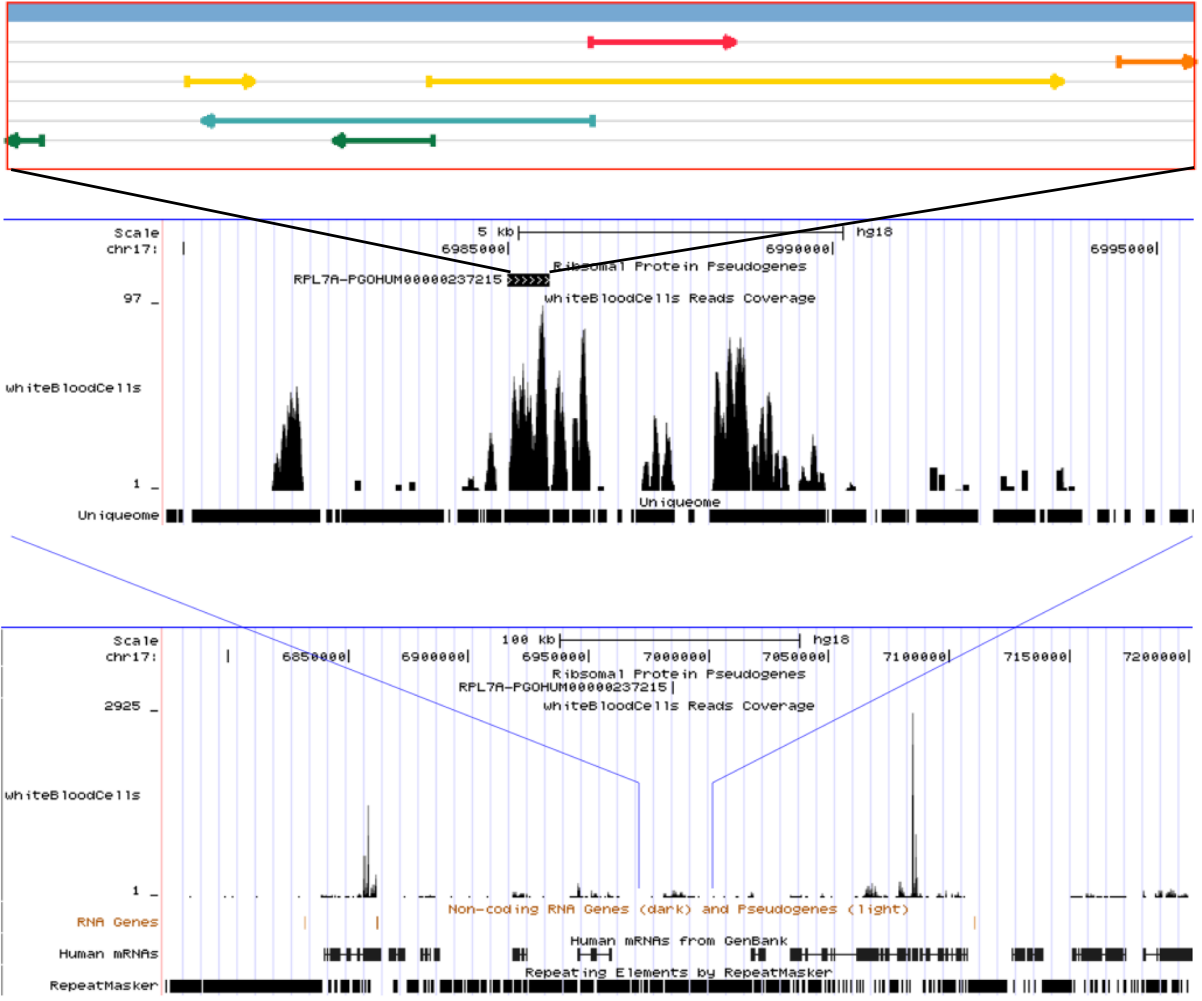
**UCSC browser view RNA-seq expression of pseudogene PGOHUM-237215 in white blood cells.** RPKM = 17, Tissue Specificity = 0.881. Open reading frames (ORFs) in +1, +2, +3, -1, -2, and −3 are annotated.

**Figure 4:**
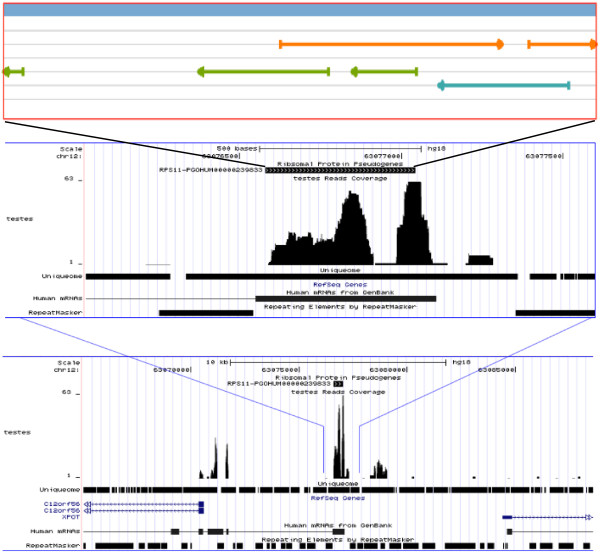
**UCSC browser view RNA-seq expression of pseudogene PGOHUM-249146 in kidney.** RPKM = 16, Tissue Specificity = 0.855. Open reading frames (ORFs) in +1, +2, +3, -1, -2, and −3 are annotated.

**Figure 5:**
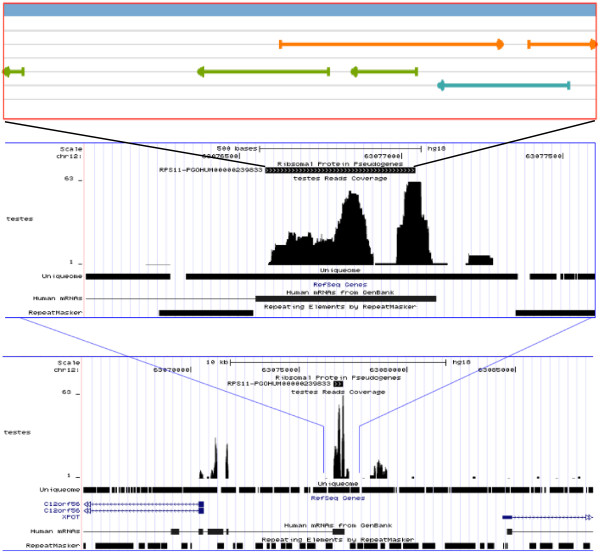
**UCSC browser view RNA-seq expression of pseudogene PGOHUM-239833 in testes.** RPKM = 11, Tissue Specificity = 0.813. Open reading frames (ORFs) in +1, +2, +3, -1, -2, and −3 are annotated.

Three additional pseudogenes are highly transcribed (RPKM > 10). **PGOHUM-237215**, an RPL7A pseudogene, is expressed RPKM = 17 in white blood cells. This pseudogene is located in an intergenic region. Also, the transcription unit seems to span a longer region (Figure 3). It is transcribed in a white blood cell specific fashion. **PGOHUM-249146**, an RPS24 pseudogene, is expressed in kidney. This pseudogene is located in the intronic region of gene SLC12A3 (Figure 4). This gene encodes a renal thiazide-sensitive sodium-chloride cotransporter that is important for electrolyte homeostasis. **PGOHUM-239833**, an RPS11 pseudogene, is expressed in testes. This pseudogene is located in an intergenic region (Figure 5). The comparison of read coverage with or without uniqueome filtering for these four RP pseudogenes can be been in Figures S1-S4 in Additional file 2.

### Tissue-specificity of pseudogene transcription

**Figure 6:**
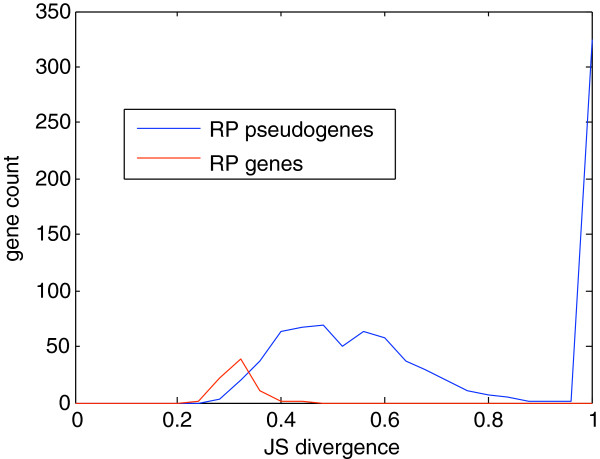
**Distribution of tissue specificity, as measured by the JS divergence**[20].

Many genes are expressed in a tissue-specific fashion. The Human Body Map 2.0 data allow us to study the tissue-specificity of transcriptions of these pseudogenes. We adopt the entropy-based Jensen-Shannon (JS) divergence measure used in [20]. The distributions of tissue-specificity JS divergences of RP pseudogenes versus RP genes are shown in Figure 6. In the Human Body Map 2.0 data set, all RP genes are not transcribed in a tissue specific fashion (JS divergence <0.5 for all RP genes) (Table S2 in Additional file 3). Unlike ribosomal protein genes that are constitutively expressed in almost all tissues, many RP pseudogenes are differentially expressed (Table S1 in Additional file 1). Among the seventeen pseudogenes with RPKM > 5 at some tissue, 8 of them have a JS divergence > 0.5. In fact, all of the top 4 pseudogenes with RPKM > 10 are transcribed in a highly tissue specific fashion (JS divergence > 0.8). These results suggest that these highly expressed RP pseudogenes may contribute to tissue-specific biological processes.

## Discussion and conclusions

In this work, we conducted a bioinformatics analysis of the pseudogenes of ribosomal protein genes in diverse human tissues. Using our specialized pipeline, we identified several cases of pseudogene expression. Most notably, one pseudogene in an intron of the TG gene is extremely highly expressed in thyroid. Moreover, several other pseudogenes are also highly expressed. We found that many pseudogenes are expressed in a tissue-specific fashion. Also, the expression of pseudogenes seems to often go beyond the boundaries of the annotated pseudogenes. Apparently, further experimental investigations will be needed to reveal the biological relevance of these expressions.

Transcriptome sequencing, RNA-seq, provides an unprecedented opportunity to uncover many complexities of cellular gene expression. A key computational challenge in RNA-seq data analysis is to discern reads among multiple potential sources with similar sequences. In this work we focused on the detection of evidences of pseudogene expression. We used extremely strict read mapping criteria to minimize potential false positives. Admittedly we did not utilize all potential reads, especially at regions with low uniqueness. Further research may consider using looser mapping criteria combined with sophisticated statistical algorithms to take into account the mapping ambiguity.

The bioinformatics methods presented here may find application in other RNA-seq studies dealing with high similarity in reference sequences. In particular, the same methodology may be able to identify differential expression between other homologous genome regions. Studies in other fields, such as metagenomics, dealing with high similarity DNA sequences may also find benefits from strict alignment and intersection with uniquely mappable locations.

## Methods

### Human tissue samples

The Human Body Map 2.0 RNA-seq data for 16 human tissue samples were provided by Gary Schroth at Illumina and can be accessible from ArrayExpress (accession no. E-MTAB-513). Reads were 75 base pairs long and came from the following samples: adipose, adrenal, brain, breast, colon, heart, kidney, liver, lung, lymph, muscle, ovary, prostate, testes, thyroid, and white blood cells. The samples were prepared using the Illumina mRNA-seq kit. They were made with a random priming process and are not stranded.

### Software and datasets

Bowtie version 0.12.7 [15] and TopHat version 1.2.0 [21] were used for the mapping. Cufflinks version 1.0.3 [17] was used for differential expression calculation for RefSeq genes. BEDTools version 2.12.0 [22] was used to analyze alignments. The uniqueome dataset was collected from the Uniqueome supplementary database [16] for human genome (hg18, NCBI Build 36.1) marking genome locations where reads of length 75 bps are unique within 4 mismatches (hg18_uniqueome.unique_starts.base-space.75.4.positive.BED). The 75 bps read length matches the RNA-seq data provided by Illumina. RefSeq genes and DNA sequences of spliced ribosomal protein genes were collected from NCBI (RefSeq database D32-6) [14]. Pseudogene annotations and sequences were downloaded from pseudogene.org [23] database (human pseudogenes build 58). Pseudogenes whose parent genes are ribosomal protein genes were selected, totaling 1788. Among them, 79 were annotated ‘Duplicated’. As we are only interested in processed pseudogenes, our analysis focuses on the remaining 1709 pseudogenes. The human genome sequence (hg18) was collected from NCBI build 36.1.

### Composite genome

A composite genome index was constructed with Bowtie-build using the sequences of the human genome (hg18, NCBI build 36.1) and NCBI spliced RP gene sequences.

### Alignment

RNA-seq data for each tissue was aligned using two distinct methodologies – one for pseudogenes and one for real genes. Pseudogene alignment protocol consists of strict alignment (Bowtie, no mismatches, report reads with only one alignment location only) to the composite genome. Real gene alignment protocol consists of strict alignment (Bowtie, no mismatches, single alignment location) to the human genome (hg18, NCBI Build 36.1).

### Uniqueome

A uniqueome data set [16] was obtained for Build 36.1 marking genome locations where reads of length 75 bps are unique within 4 mismatches. Alignments for all tissues for both real genes and pseudogenes were intersected with the uniqueome dataset for all genome locations (intersectBed from BEDTools [22]). The total number of remaining reads in each alignment was counted. The uniqueome dataset was used to filter out ambiguously mapped reads.

### Comparative expression analysis

Gene expression values were calculated as reads aligned to gene per kilobase of exon per million mapped reads (RPKM) [11]. The number of reads aligned to all gene exons and additionally aligning in unique locations was counted for each gene. Exon length for genes was calculated as the sum of unique positions as marked by the uniqueome across all gene exons. It is worth noting that RP pseudogenes appear spliced in the human genome and therefore have only a single exon for counting aligned reads and calculating exon length.

Expression percentiles of RefSeq genes were calculated using TopHat to map reads to the human genome (hg18, NCBI build 36.1) and Cufflinks was used to calculate FPKM values of all 37,681 RefSeq genes. Expression percentiles were calculated for specific tissues and for all datasets combined.

Gene reads coverage was calculated using the coverageBed program in the BEDTools software suite. Coverage represents the fraction of RP pseudogene exon covered by reads that aligned to unique genome regions.

### Tissue-specificity analysis

We followed the definition of Jensen-Shannon divergence in [20]. To avoid zero probabilities, all RPKM numbers are added by 10^-10^.

## Competing interests

The authors declare that they have no competing interests.

## Authors’ contributions

PT and VS carried out the bioinformatics analyses. PT, SZ, and DZ drafted the manuscript. SZ and DZ designed the composite genome method. DZ conceived of the study. All authors read and approved the final manuscript.

## Supplementary Material

Additional file 1 **Table S1.** RPKM expression values of RP pseudogenes in all 16 tissues. Click here for file

Additional file 2 **Figure S1-S4.** Comparison of read coverage with or without uniqueome filtering for four RP pseudogenes. Click here for file

Additional file 3 ** Table S2.** RPKM expression values of RP genes in all 16 tissues. Click here for file
