# Supplementary material for: Detecting transcription of ribosomal protein pseudogenes in diverse human tissues from RNA-seq data
Source: BMC Genomics. 2012 Aug 21;13:412. doi: 10.1186/1471-2164-13-412 (PMC3478165; doi:10.1186/1471-2164-13-412)
Supplement: Additional file 2 — Figure S1-S4. Comparison of read coverage with or without uniqueome filtering for four RP pseudogenes. [file 1471-2164-13-412-S2.doc]

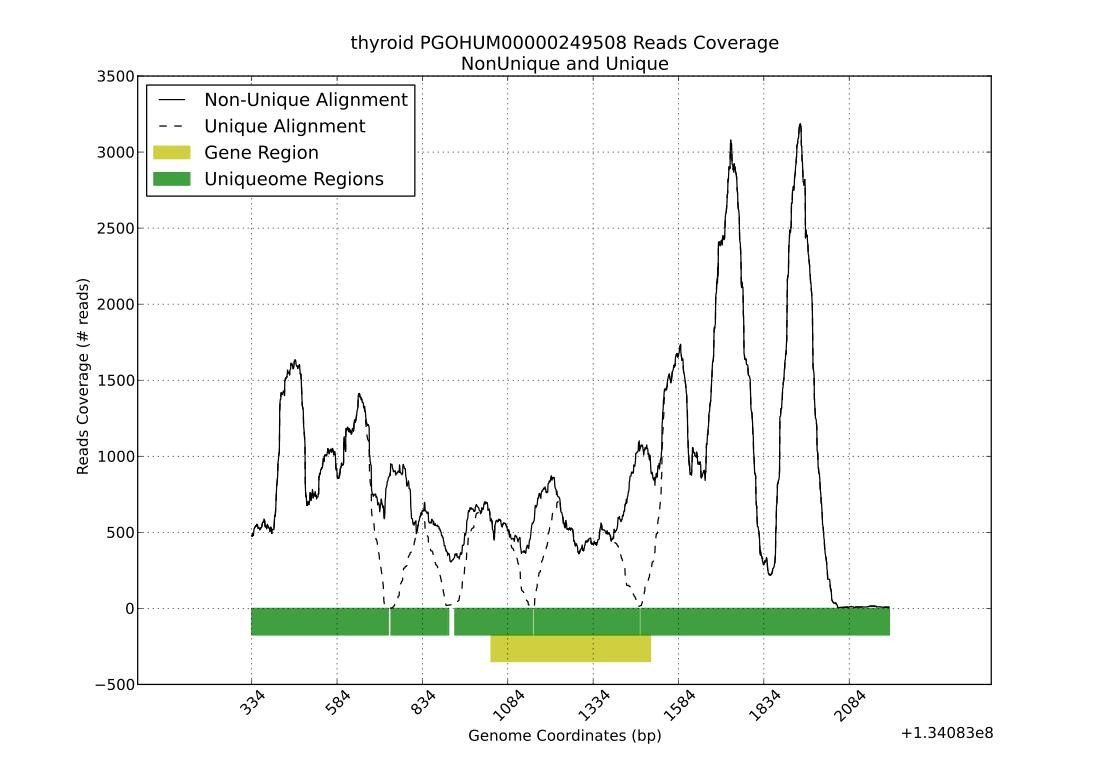


Figure S1: Comparison of read coverage with or without uniqueome filtering for pseudogene PGOHUM00000249508 in Thyroid.


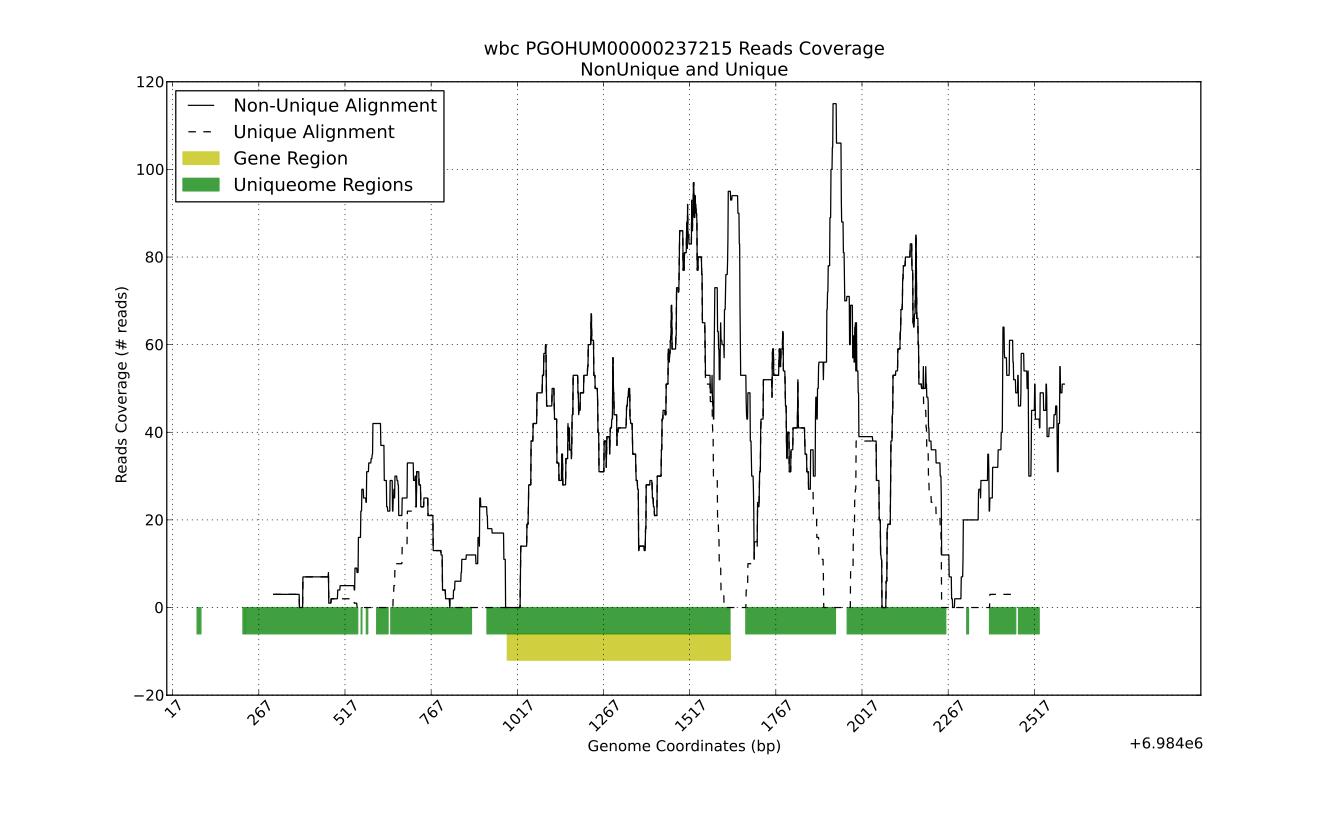
Figure S2: Comparison of read coverage with or without uniqueome filtering for pseudogene PGOHUM00000237215 in white blood cells.


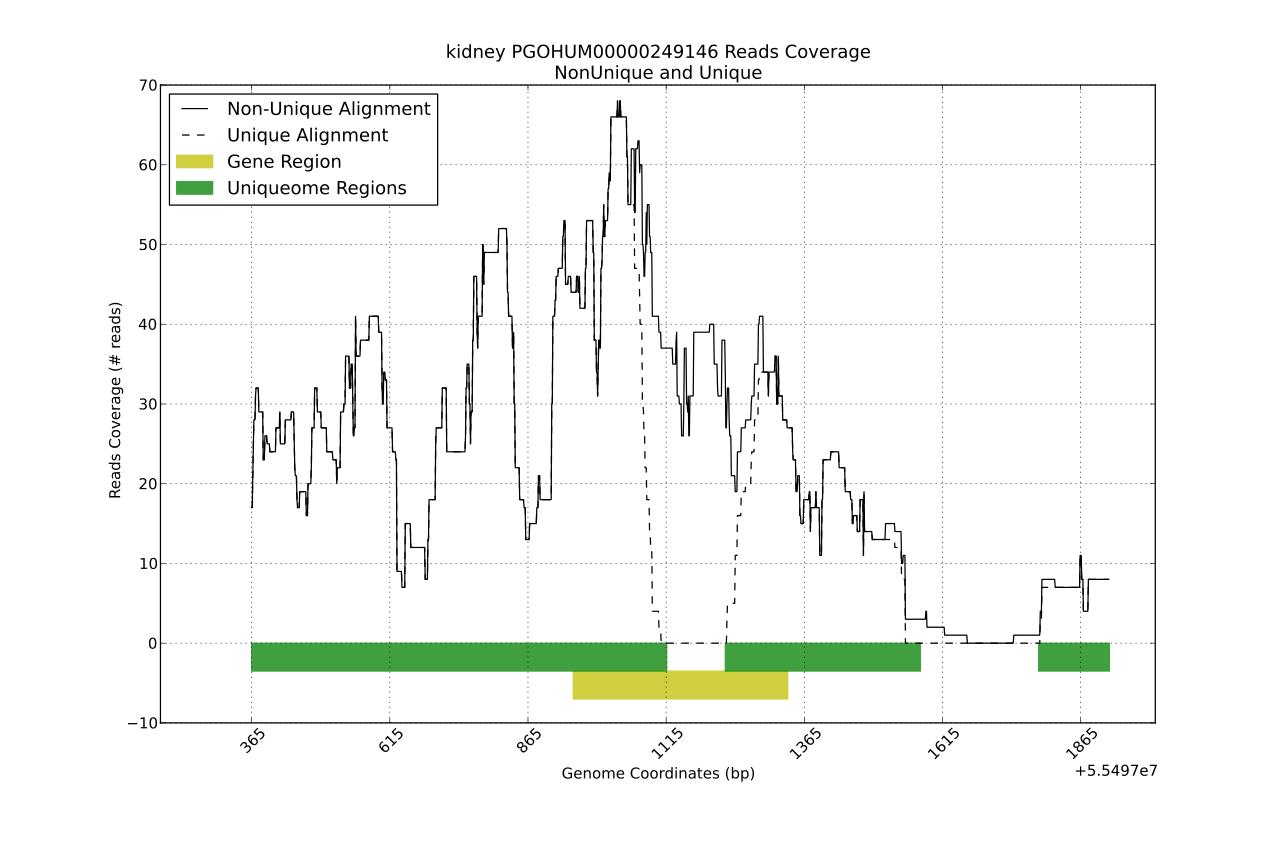


Figure S3: Comparison of read coverage with or without uniqueome filtering for pseudogene PGOHUM00000249146 in kidney.


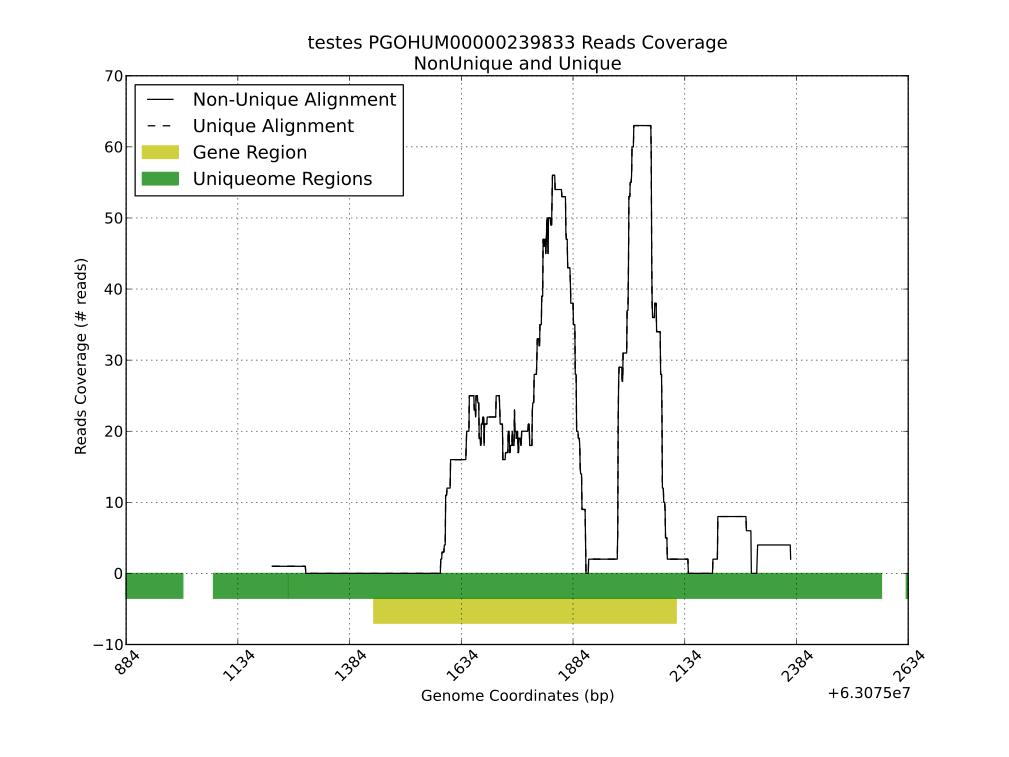


Figure S4: Comparison of read coverage with or without uniqueome filtering for pseudogene PGOHUM00000239833 in testes.
